# Key Common Genes with LTF and MMP9 Between Sepsis and Relapsed B-Cell Lineage Acute Lymphoblastic Leukemia in Children

**DOI:** 10.3390/biomedicines13092307

**Published:** 2025-09-20

**Authors:** Ying-Ping Xiao, Yu-Cai Cheng, Chun Chen, Hong-Man Xue, Mo Yang, Chao Lin

**Affiliations:** 1 Pediatric Hematology Laboratory, Division of Hematology/Oncology, Department of Pediatrics, The Seventh Affiliated Hospital, Sun Yat-sen University, Shenzhen 518107, China; xiaoyingping@sysush.com (Y.-P.X.); chengyucai@sysush.com (Y.-C.C.); chenchun@mail.sysu.edu.cn (C.C.); xuehm5@mail.sysu.edu.cn (H.-M.X.); 2 Scientific Research Center, The Seventh Affiliated Hospital, Sun Yat-sen University, Shenzhen 518107, China

**Keywords:** common genes, sepsis, relapsed B-ALL, LTF, MMP9

## Abstract

**Background:** Pediatric sepsis is a life-threatening disease that is associated with the progression of acute lymphoblastic leukemia (ALL) and the recurrence of B-cell ALL (B-ALL). Although previous studies have reported a partial association between sepsis and ALL, there is limited research on the shared genes between pediatric sepsis and relapsed B-ALL. This study aims to further elucidate the more comprehensive and novel common genetic factors and molecular pathways between the two diseases. **Methods:** Gene expression datasets pertaining to pediatric sepsis (GSE13904, GSE80496) and relapsed B-ALL (GSE3910, GSE28460) were retrieved from the Gene Expression Omnibus database for this retrospective analysis. The initial analysis identified differentially expressed genes common to both pediatric sepsis and relapsed B-ALL. Subsequent investigations employed three complementary approaches: protein–protein interaction networks, molecular complex detection (MCODE) clustering functions, and support vector machine recursive feature elimination model to separately identify the diagnostic biomarkers for each condition. Importantly, key common genes were identified by overlapping the diagnostic genes for pediatric sepsis and relapsed B-ALL. Further characterization involved comprehensive functional analysis through the Metascape platform, construction of transcription factor (TF)-mRNA-microRNA (miRNA) networks, drug prediction, and molecular docking to explore their biological significance and potential therapeutic targets. **Results:** Comparative analysis of pediatric sepsis-related and relapsed B-ALL-related datasets revealed two shared genetic markers, lactotransferrin (LTF) and matrix metallopeptidase 9 (MMP9), exhibiting diagnostic significance and consistent upregulation in both disease groups. Transcriptional regulatory network analysis identified specificity protein 1 (SP1) as the principal transcription factor capable of coregulating LTF and MMP9 expression. In addition, molecular docking demonstrated high-affinity interactions between curcumin and MMP9 (−7.18 kcal/mol) as well as reserpine and LTF (−5.4 kcal/mol), suggesting their potential therapeutic utility for clinical evaluation. **Conclusions:** These findings elucidate the molecular pathogenesis involving LTF and MMP9 in pediatric sepsis and relapsed B-ALL, providing novel insights for clinical diagnosis and therapeutic development.

## 1. Introduction

B-cell acute lymphoblastic leukemia (B-ALL) constitutes approximately 85% of pediatric acute lymphoblastic leukemia (ALL), representing the most prevalent childhood cancer that accounts for 25% of all pediatric malignancies [1]. Over the last ten years, treatment advancements such as chemotherapy, immunotherapy, targeted therapy, and hematopoietic stem cell transplantation (HSCT) have achieved cure rates approaching 90% in pediatric ALL populations [2]. However, disease recurrence persists as a major clinical challenge, occurring in 15–20% of cases and contributing substantially to treatment failure [3]. The identification of efficacious biomarkers for relapsed B-ALL is crucial in facilitating the exploration and enhancement of therapeutic interventions for this disease.

Sepsis is a potentially fatal condition characterized by organ failure, primarily resulting from an imbalanced reaction of the body to an infection [4]. Global epidemiological data indicate that approximately 1.2 million cases of sepsis worldwide are diagnosed each year [5]. Pediatric populations exhibit particularly high mortality rates ranging from 25% to 50% following sepsis hospitalization [6]. Both sepsis and relapsed ALL constitute severe threats to the life and health of children. CD19-targeted chimeric antigen receptor (CAR) T-cell therapy has demonstrated potential to improve prognosis in relapsed or drug-resistant ALL, though this intervention carries an increased sepsis risk that may subsequently exacerbate ALL progression [7]. Notably, pediatric leukemia patients receiving intensive chemotherapy regimens or hematopoietic stem cell transplantation show markedly heightened susceptibility to severe sepsis development [8]. Pathogenesis involves three primary mechanisms contributing to infection susceptibility in leukemia patients. Firstly, leukemia itself and its treatments cause both quantitative and qualitative deficiencies in key immune cells, such as neutrophils and lymphocytes, thereby compromising anti-infection defenses [9,10]. Secondly, chemotherapy regimens disrupt intestinal epithelial integrity, facilitating bacterial translocation that increases the risk of systemic infection and sepsis [11,12]. Additionally, the leukemia-associated proinflammatory milieu primes patients for exaggerated cytokine responses during infections, accelerating progression to severe sepsis and organ dysfunction [13]. This bidirectional detrimental association between relapsed B-ALL and sepsis highlights the critical importance of investigating their shared molecular mechanisms. Therefore, further identification of common key genes and exploration of their biological functions and regulatory networks are anticipated to provide novel perspectives for the clinical diagnosis and development of therapeutic targets for pediatric sepsis and relapsed B-ALL.

Previous bioinformatics analyses of pediatric ALL and pediatric sepsis cases have revealed three shared key genes (ring finger protein 125 (RNF125), noggin (NOG), and hemopoietic cell kinase (HCK)) [14]. However, these analyses did not focus on specific subtypes of ALL, such as relapsed B-ALL. Clinically, relapsed B-ALL is associated with poorer prognosis, and its correlation with sepsis holds greater clinical urgency. Furthermore, the findings of this prior study have not been validated using clinical samples. To date, no research has yet investigated the shared genes between relapsed B-ALL and sepsis. The current work represents the first systematic investigation into the intersecting pathogenic pathways of sepsis and relapsed B-ALL. Through analysis of transcriptomic profiles from the Gene Expression Omnibus (GEO) database, incorporating datasets from both pediatric sepsis infections and relapsed B-ALL cases, common differentially expressed genes were identified and their biological roles. Furthermore, transcriptional regulatory networks incorporating transcription factors (TFs), mRNAs, and microRNAs (miRNAs) were constructed, revealing novel molecular interactions that may contribute to disease pathogenesis. Finally, we validated the expression patterns of the target genes in clinical samples. This work establishes a theoretical foundation and identifies potential therapeutic targets for advancing the understanding of the common pathogenic mechanisms in pediatric sepsis and relapsed B-ALL.

## 2. Materials and Methods

### 2.1. Data Extraction

In this study, gene expression data of pediatric sepsis and healthy controls (HC), as well as bone marrow samples of B-ALL at initial diagnosis and relapse, were downloaded from the GEO. We chose the included datasets based on three core principles: sample relevance to research objectives, sufficient sample size for robust analysis, and reliable data quality with detailed clinical metadata—all to ensure alignment with our goal of identifying shared genes between pediatric sepsis and relapsed B-ALL:

For pediatric sepsis, the analysis incorporated two gene expression datasets for their blood-derived samples (consistent with the accessible clinical sample type for sepsis) and clear case–control design (sepsis vs. HC): GSE13904 (GPL570 platform) served as the training set due to its larger sample size (35 pediatric septicemia blood samples on day 1 and 18 HC samples) [15]. For validation purposes, GSE80496 (GPL6883 platform) provided 21 pediatric septicemia samples, and 21 HC blood samples; 3 meningitic septicemia samples were excluded to ensure the homogeneity of sepsis subtypes [16]. Both datasets avoided non-blood samples that are less clinically relevant for sepsis screening, and their sample sizes (exceeding 8 cases/controls per group) met the minimum requirement for stable differential expression analysis.

For relapsed B-ALL-related research, GSE3910 (GPL96 platform) [3] and GSE28460 (GPL570 platform) [17] were included because they exclusively provided bone marrow samples (the gold-standard tissue for B-ALL diagnosis/relapse assessment) and directly compared relapsed vs. initially diagnosed B-ALL (GSE3910 act as the training set: 35 relapsed + 35 initial cases; GSE28460 act as the validation set: 49 relapsed + 49 initial cases). This design directly addressed our focus on “relapsed” B-ALL (a subtype understudied in prior sepsis-ALL association research).

Both datasets underwent log2 transformation prior to analysis. And all selected datasets were downloaded from public database with detailed metadata (e.g., instructions and guidelines, clinically sample source that enabled comprehensive investigation of gene changes, and clinical diagnosis information) and easy accessibility, facilitating result verification. Dataset exclusion criteria included insufficient sample size, incomplete clinical metadata, or detectable data quality issues to avoid bias. Additionally, our dataset selection and analysis methodology adheres to Standards for Reporting of Diagnostic Accuracy (STARD) guidelines [18].

### 2.2. Screening for the Differentially Expressed Common Genes in Pediatric Sepsis and Relapsed B-ALL

Firstly, the differentially expressed genes 1 (DEGs1) between 32 pediatric sepsis and 18 HC samples in the GSE13904 dataset, and DEGs2 between 35 pediatric B-ALL (initial diagnosis) and 35 pediatric B-ALL with marrow relapse samples in the GSE3910 dataset were identified with the “limma” R package (version 3.52.4) (|log2FoldChange (FC)| > 0.5, *p* < 0.05) [19,20,21], respectively. Then, the common genes were obtained by intersecting the DEGs1 and DEGs2 using the “ggvenn” R package (version 1.7.3).

### 2.3. Functional Enrichment Analysis of Common Genes

Functional enrichment analysis of Gene Ontology (GO) functions and Kyoto Encyclopedia of Genes and Genomes (KEGG) pathways for these common genes was conducted with the “clusterprofiler” R package (version 4.2.2) (*p* < 0.05) [22].

### 2.4. Screening for the Diagnostic Genes of Pediatric Sepsis and Relapsed B-ALL

In this study, the protein–protein interaction (PPI) network was constructed to investigate the relationships of the above common genes by the “Retrieval of Interacting Gene/Proteins (STRING) database” database (https://string-db.org/, accessed on 2 September 2023), and the hub genes were screened using “MCODE” [23]. Then, the diagnostic genes of pediatric sepsis and relapsed B-ALL were screened by the support vector machine recursive feature elimination (SVM-RFE) method with 5-fold cross validation, respectively. In addition, the receiver operating characteristic (ROC) curves were drawn to assess the diagnostic ability of diagnostic genes both in the training and validation datasets. The expression of diagnostic genes between two groups in the different datasets were compared by Wilcoxon test (*p* < 0.05).

### 2.5. Expression and FUNCTION Analysis of the Key Common Genes of Pediatric Sepsis and Relapsed B-ALL

Based on above analyses, the expression levels of key common genes that obtained by intersecting these two groups diagnostic genes were calculated in all training and validation datasets. GO functions of these key common genes were performed in Metascape database by “clusterProfiler” R package (version 4.2.2).

### 2.6. Molecular Mechanism Analyses

The regulatory network between transcription factors (TFs), mRNAs and microRNAs (miRNAs) is one of the important mechanisms for the regulation of gene expression. This network describes the interactions between TFs, mRNAs and miRNAs and how they work together to regulate gene expression. As the perspective of molecular mechanism, the targeted TFs were predicted in TRRUST database, and the targeted miRNAs were predicted in miRNet database. Then, the TF-mRNA-miRNA regulatory network of the key common genes was constructed by “Cytoscape” (version 3.8.2) [24].

### 2.7. Drug Prediction and Molecular Docking

The targeted drugs of key common genes were predicted in the Drug-Gene Interaction database (DGIdb), and the interaction relationships between targeted drugs and key common genes were visualized by “Cytoscape” (version 3.8.2). Furthermore, the protein structure of genes was downloaded from the Protein Data Bank (PDB), and the structure of key drugs were downloaded from “PubChem”. Then, virtual screening of drugs was performed through molecular docking using AutoDockvina, and the inter-molecular interaction between gene and drug were explored by this study.

### 2.8. Real Time Quantitative PCR (RT-qPCR)

A total of peripheral blood samples from patients with 5 relapsed B-ALL with sepsis and 5 controls [25] were collected from the Seventh Affiliated Hospital, Sun Yat-Sen University. Ethical approval for this study was granted by Ethics Committee of Seventh Affiliated Hospital, Sun Yat-Sen University (KY-2025-008-01). Written informed consent was procured from all participants or their legally authorized representatives prior to study enrollment. Total RNA was extracted from the samples using the FastPure Complex Tissue/Cell Total RNA Isolation Kit (Vazyme, Nanjing, China), with the A260/A280 ratio falling within the range of 1.8–2.0. Reverse transcription was carried out using the ABScript III RT Master Mix for qPCR with gDNA Remover (ABclonal, Wuhan, China). RT-qPCR was performed with the Genious 2X SYBR Green Fast qPCR Mix (ABclonal, Wuhan, China). Each sample was analyzed in triplicate. GAPDH was used as the reference gene, and the relative mRNA expression levels were compared using the 2^−ΔΔCt^ method. Primer sequences for signature genes were designed and indicated in Table 1.

### 2.9. Statistical Analysis

All analyses were conducted using R language (https://www.r-project.org/, accessed on 2 September 2023). Differences between two groups were compared by “Wilcoxon” test. If not specified above, *p* < 0.05 was regarded as statistically significant.

## 3. Results

### 3.1. The 164 Common Genes in Pediatric Sepsis and Relapsed B-ALL Were Related to Inflammation and Immune Response

**Figure 1:**
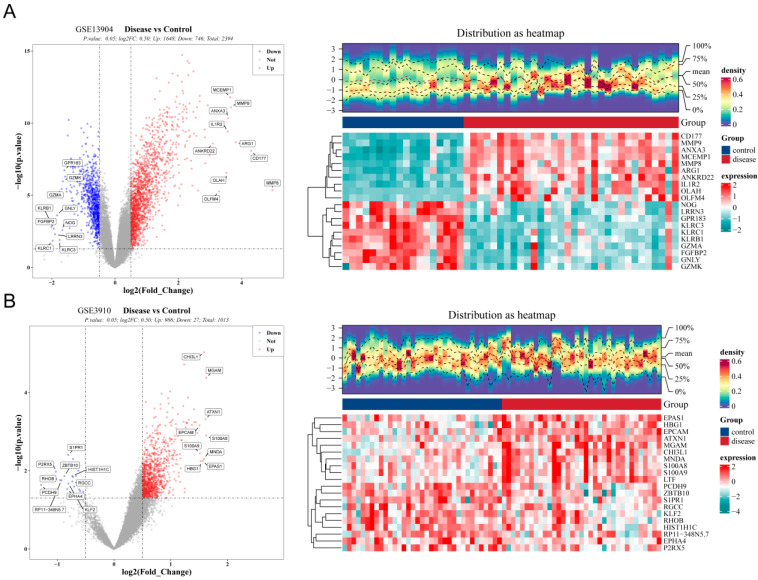
Identification of differentially expressed common genes in pediatric sepsis and relapsed B-ALL. (**A**) In the GSE13904 dataset, differentially expressed genes 1 (DEGs1) was identified between pediatric sepsis and healthy controls (HC) samples. The criteria were |log2FC| > 0.5 and *p* < 0.05. The left panel shows a volcano plot of DEGs1, where red dots represent upregulated genes and blue dots represent downregulated genes. The right panel displays a heatmap of the top 10 upregulated and downregulated DEGs1. Each column represents a sample, each row represents a gene, with red indicating high expression and blue indicating low expression. (**B**) In the GSE3910 dataset, differentially expressed genes 2 (DEGs2) was identified between pediatric B-ALL at initial diagnosis and pediatric B-ALL with bone marrow relapse. The criteria were |log2FC| > 0.5 and *p* < 0.05.

**Figure 2:**
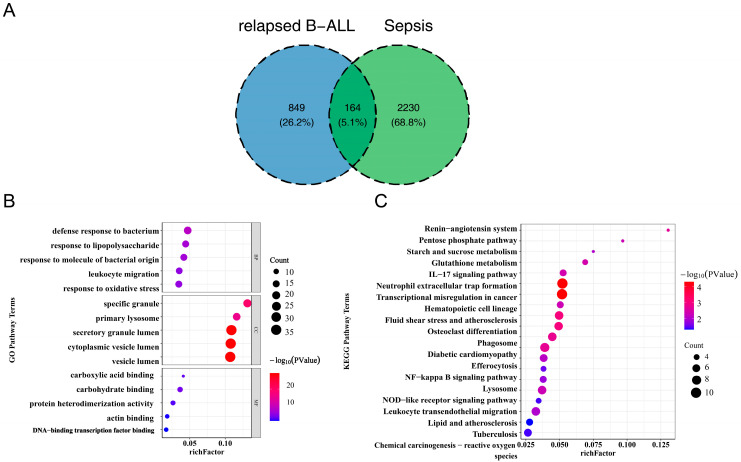
Identification and functional analysis of common DEGs. (**A**) Venn diagrams represent DEGs common to pediatric sepsis and relapsed B-ALL. (**B**) Gene Ontology (GO) enrichment analysis of the common DEGs. The enrichment factor was plotted along the abscissa, while the ordinate displayed GO terms categorized into biological processes, cellular components, and molecular functions. Bubble dimensions indicated the count number, and the color represented the significance. (**C**) Kyoto Encyclopedia of Genes and Genomes (KEGG) enrichment analysis of the common DEGs. The abscissa represented the enrichment factor, and the ordinate represented the KEGG pathways. Note: B-ALL, B-cell lineage acute lymphoblastic leukemia.

In this study, differential expression analysis identified 2394 DEGs1, including 1648 upregulated genes and 746 downregulated genes, as well as 1013 DEGs2, which comprised 986 upregulated genes and 27 downregulated genes (Figure 1A,B, Table S1). Subsequently, by taking the intersection of DEGs1 and DEGs2, a total of 164 common genes associated with pediatric sepsis and relapsed B-ALL were identified (Figure 2A). Functional enrichment analysis revealed that these common genes were enriched in 518 GO terms with statistical significance (*p* < 0.05) (Figure 2B, Table S2). Specifically, 414 biological process (BP) terms were identified, such as defense response to bacterium and response to lipopolysaccharide (LPS). Additionally, 48 cellular component (CC) terms were noted, such as specific granule and primary lysosome. Furthermore, 56 molecular function (MF) terms were identified, such as carboxylic acid binding and carbohydrate binding.

Notably, KEGG pathway analysis indicated that the 164 common genes were enriched in 23 pathways (*p* < 0.05) (Figure 2C, Table S3), with the IL-17 signaling pathway standing out as a core intersectional pathway—directly relevant to the pathological progression of both pediatric sepsis and relapsed B-ALL and have been well documented to drive immunopathology, autoimmune disease, and cancer progression [26,27]. For pediatric sepsis, recent studies have explicitly identified the IL-17 signaling pathway as a key mediator of disease severity including recruiting neutrophils to infected tissues, thereby exacerbating organ dysfunction [28]. In the context of relapsed B-ALL, IL-17A activates the Akt signaling pathway to promote resistance to daunorubicin, reducing treatment efficacy in relapsed cases [29]. Additionally, pathways including starch and sucrose metabolism, the renin-angiotensin system, the pentose phosphate pathway, and glutathione metabolism were also enriched. Notably, a substantial proportion of these pathways exhibited close associations with immune response, inflammatory response, and oxidative stress, suggesting that pediatric sepsis and relapsed B-ALL shared similarities in immune system function and regulation, and might mediate their pathological processes through comparable immune mechanisms.

### 3.2. 4 Diagnostic Genes of Sepsis and 2 Diagnostic Genes of Relapsed B-ALL Were Screened

**Figure 3:**
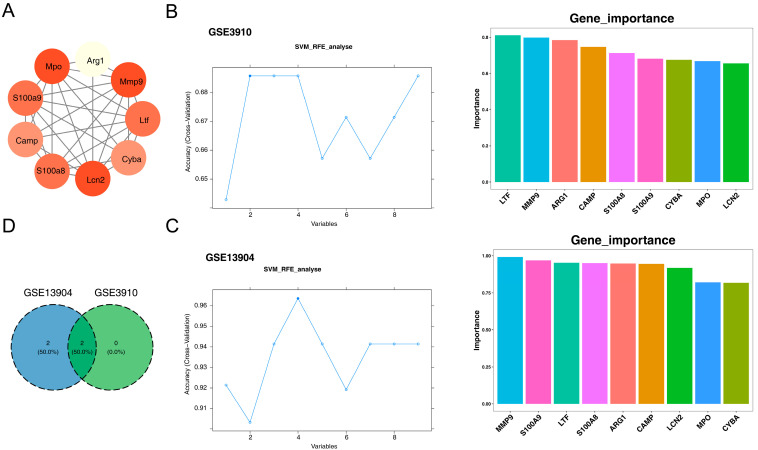
Identification and evaluation of the diagnostic genes for pediatric sepsis and relapsed B-ALL. (**A**) Nine hub genes were selected using “MCODE”. Different circles represent different genes, and the connecting lines indicate interaction relationships between the proteins expressed by these genes. The darker the color of the circle, the more interaction relationships it has. (**B**) Two diagnostic genes for relapsed B-ALL were identified by SVM-RFE method. On the left is a plot of accuracy against the number of variables, and on the right is a bar chart showing the importance of genes. (**C**) Four diagnostic genes for pediatric sepsis were detected by SVM-RFE method. (**D**) Venn diagram of diagnostic genes (LTF and MMP9) for the two disease datasets. Note: SVM-RFE, support vector machine recursive feature elimination.

The Protein–protein interaction (PPI) network of above common genes was constructed and 9 hub genes (CAMP, LCN2, LTF, MMP9, MPO, S100A8, S100A9, Arg1, Cyba) were obtained (Figure 3A, Figure S1). Then, four diagnostic genes of sepsis (LTF, MMP9, S100A9, and S100A8) and two diagnostic genes of relapsed B-ALL (LTF and MMP9) were screened by SVM-RFE, respectively (Figure 3B,C). The diagnostic genes selected from two disease datasets were intersected to obtain two Hub genes, namely MMP9 and LTF (Figure 3D).

### 3.3. LTF and MMP9 Were the Key Common Genes of Pediatric Sepsis and Relapsed B-ALL

**Figure 4:**
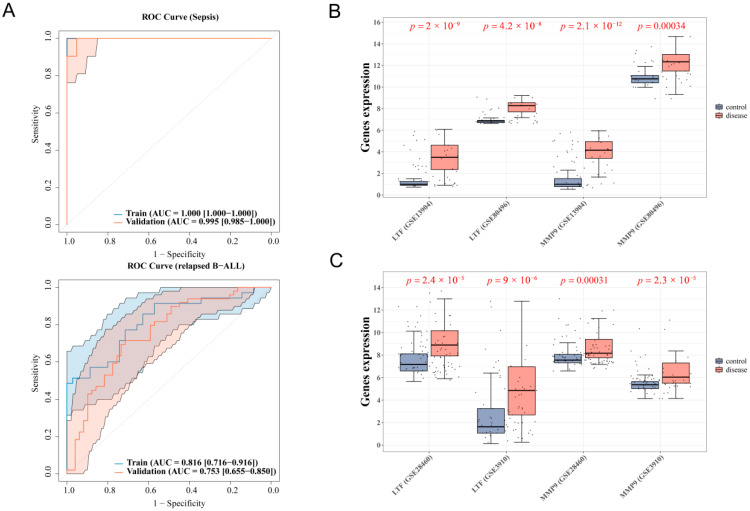
Predictive performance and expression of two key genes common to pediatric sepsis and relapsed B-ALL. (**A**) ROC curves of two key common genes (pediatric sepsis in GSE13904 and GSE80496, and relapsed B-ALL in GSE3910 and GSE28460). (**B**) Boxplot for expression of two key common genes in training and validation datasets of children sepsis. (**C**) Boxplot for expression of two key common genes in training and validation datasets of relapsed B-ALL. Note: ROC, receiver operating characteristic; AUC, area under the curve.

Based on the four diagnostic genes for sepsis and two diagnostic genes for relapsed B-ALL, LTF and MMP9 were identified as two key common genes. In pediatric sepsis cohorts, the combined analysis of LTF and MMP9 achieved perfect classification (the area under the curve [AUC] = 1.0) in training set GSE13904, with near-perfect validation (AUC = 0.995) in the validation set GSE80496. For relapsed B-ALL, the biomarkers demonstrated robust diagnostic accuracy, yielding an AUC value of 0.816 in the training set GSE3910, and 0.753 in the validation set GSE28460. All AUC values exceeded 0.7, indicating that LTF and MMP9 had significant diagnostic value for both pediatric sepsis and relapsed B-ALL (Figure 4A). Differential gene expression analysis further demonstrated marked transcriptional upregulation of both LTF and MMP9 in the sepsis and relapsed B-ALL groups relative to the control group (*p* < 0.05) (Figure 4B,C), further supporting their potential as diagnostic biomarkers. In addition, the expression of LTF and MMP9 in the GSE26378 were consistent with the expression of them in the GSE13904 dataset, and the AUC of them were all greater than 0.7 (Figure S3).

### 3.4. The Key Common Genes Were Associated with the Pathogenesis of Sepsis and Relapsed B-ALL

**Figure 5:**
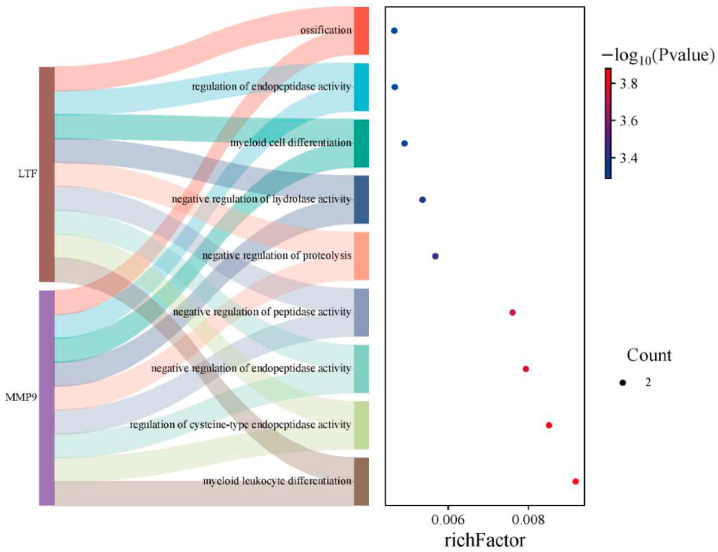
Functional enrichment analysis of shared genes using Metascape-derived GO annotations. The Sankey diagram for GO functions, with node size corresponding to gene count and color reflecting statistical significance (−log10 [*p*-value]), where deeper red hues indicate greater enrichment significance.

GO analysis identified significant functional associations between the core common genes and four biological processes: inhibition of peptidase activity, negative regulation of endopeptidase activity, regulation of cysteine-type endopeptidase activity, and myeloid leukocyte differentiation (Figure 5). Notably, these molecular pathways demonstrate direct relevance to the disease mechanisms underlying both sepsis and relapsed B-ALL.

### 3.5. Molecular Mechanism Analyses of Key Common Genes

**Figure 6:**
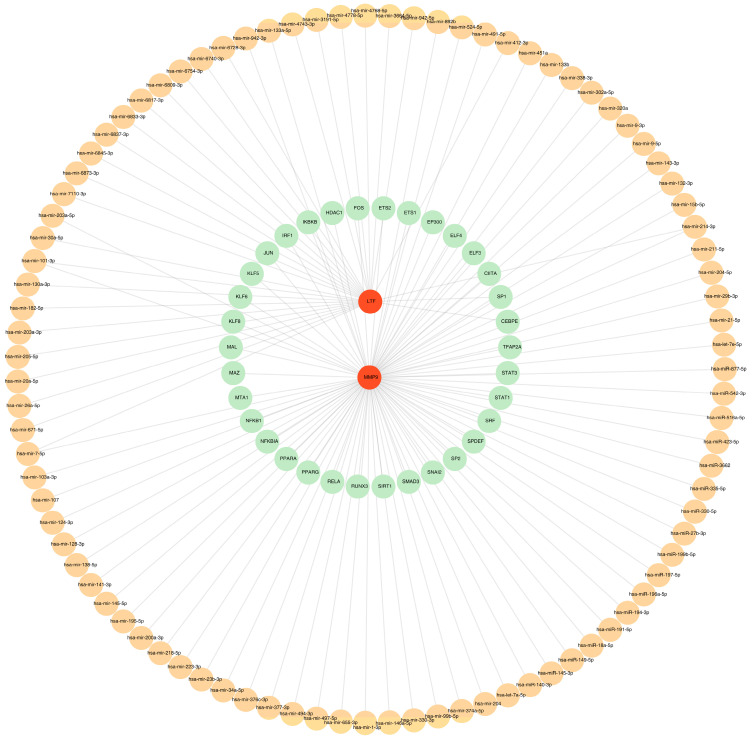
The underlying regulatory network targeting two key common genes. Red represents gene, orange represents miRNA, green represents transcription factors (TFs).

In this study, the TF-mRNA-miRNA regulatory network of key common genes were constructed with 35 TFs and 94 miRNAs (Figure 6, Figure S2). In this network, hsa-mir-214-3p, hsa-mir-101-3p, and hsa-mir-7-5p were the common miRNAs of LTF and MMP9. SP1 was the key TF which could regulate LTF and MMP9 at the same time.

### 3.6. Prediction of MMP9/LTF-Targeted Drugs and Molecular Docking Validation

**Figure 7:**
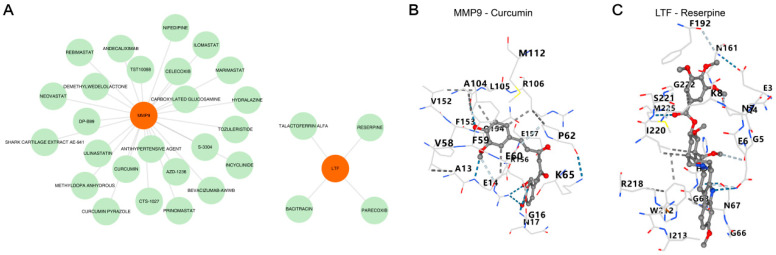
Identification of potential drugs targeting two key common genes and molecular docking. (**A**) The drug-gene interaction network through DGIdb database. LavenderBlush represents gene, Green represents drug. (**B**) Molecular docking results of MMP9 and *Curcumin* using AutoDockvina. (**C**) Molecular docking results of LTF and Reserpine using AutoDockvina. Red indicates oxygen atoms, gray indicates carbon atoms, and blue dashed lines indicate hydrogen bonds.

In addition, totals of 25 targeted drugs of MMP9 and 4 targeted drugs of LTF were predicted (Figure 7A). Among them, there were five hydrogen bonds between MMP9 and Curcumin, and the docking affinity was −7.18 kcal/mol. The docking affinity between LTF and Reserpine was −5.4 kcal/mol (Figure 7B,C). It indicated that there was a strong binding affinity between the MMP9 protein and the small molecule Curcumin, as well as between the LTF protein and the small molecule Reserpine, forming potent compound-target pairs. Notably, the findings pertaining to Curcumin and Reserpine are purely in silico predictions, derived exclusively from molecular docking simulations using AutoDockvina (version 1.2.0). These results remain theoretical and require validation with prior experimental evidence.

### 3.7. Validation of the Expression of Common Genes in the Relapsed B-ALL and Sepsis

**Figure 8:**
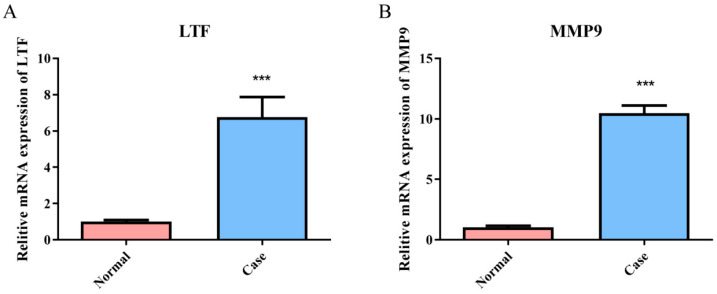
RT-qPCR analysis revealed significant differences in the mRNA expression levels of LTF (**A**) and MMP9 (**B**) between the relapsed B-ALL with sepsis and control groups. ***, *p* < 0.001.

The expression of common genes in B-ALL and sepsis was explored via RT-qPCR. The results indicated that the expression levels of LTF and MMP9 in the relapsed B-ALL with sepsis group were significantly higher than those in the control group (*p* < 0.001) (Figure 8A,B).

## 4. Discussion

Severe infections critically modify therapeutic strategies for pediatric ALL cases due to heightened sepsis susceptibility stemming from patient-specific and therapeutic factors [30]. Such infectious complications frequently necessitate chemotherapy delay, consequently increasing the relapse rate of ALL [31]. Molecular investigations establish correlations between ALL relapse and dysregulated gene expression patterns [32], while genetic and epigenetic alterations play an important role in the diagnosis and clinical management [33]. However, whether there is a shared gene between relapsed ALL and sepsis remains unclear. Utilizing bioinformatic approaches, our study newly identified LTF and MMP9 as shared hub genes in pediatric sepsis and relapsed B-ALL. Both genes demonstrated significant upregulation in disease cohorts and showed excellent combined diagnostic performance. The systematic construction of a TF-miRNA-mRNA regulatory network identified SP1 as a pivotal transcriptional regulator of these targets, while simultaneously predicting curcumin and reserpine as potential therapeutic agents. These findings not only elucidate novel shared molecular mechanisms between these conditions but also yield clinically relevant biomarker candidates for diagnostic applications.

In this study, comparative transcriptomic analysis identified 164 DEGs common to both pediatric sepsis and relapsed B-ALL cases. Functional enrichment analysis revealed predominant involvement of these genes in the regulation of inflammatory and immune responses, particularly highlighting significant enrichment within the IL-17 signaling pathway. The IL-17 family, comprising six structurally similar members (IL-17A to IL-17F), has garnered substantial research attention given the well-characterized proinflammatory role of its prototypical member IL-17A in autoimmune diseases [34]. Experimental evidence from Gan et al. illustrates that IL17-RA-1 mitigates the sepsis response through miR-7847-3p/MAPK pathway modulation [35], implying therapeutic potential for this competing endogenous RNA (ceRNA) network in both sepsis prediction and intervention strategies. Furthermore, Bi et al. have shown that IL-17A fosters resistance to daunorubicin by activating the Akt signaling pathway, and inhibitors of PI3K/Akt such as LY294002 or perifosine can effectively restore daunorubicin-induced cell death in relapsed B-ALL cells [29]. The present findings collectively indicate a potential association between IL-17A and the progression of both sepsis and relapsed B-ALL. Targeting the IL-17A pathway or its downstream signaling represents a promising therapeutic opportunity for mitigating pathological advancement in these clinical contexts. However, it must be noted that these hypotheses originate from integrative bioinformatic assessments coupled with existing literature evidence. Subsequent experimental validation remains imperative to substantiate these mechanisms and comprehensively evaluate the clinical applicability of IL-17A pathway inhibition in these diseases.

Comparative genomic analysis identifies LTF and MMP9 as key overlapping genes implicated in both pediatric sepsis and relapsed B-ALL. This finding implies conserved functional roles and regulatory networks across these clinically distinct conditions. Research further indicates that elevated LTF concentrations in septic patients further support its potential involvement in disease mechanisms [36], and LTF mitigates the systemic inflammatory response by binding to LPS and inhibiting the release of inflammatory mediators [37]. Notably, due to the immunosuppression induced by ALL therapy, patients with B-ALL are highly susceptible to infections [38]; here, LTF’s demonstrated antimicrobial efficacy could help reduce infection susceptibility during chemotherapy-induced immunosuppressive states. Beyond its anti-inflammatory and antimicrobial effects, this multifunctional protein also exhibits tissue-protective effects through inflammatory microenvironment regulation [39]. Consistent with these roles, LTF expression was significantly upregulated in children with multiple organ dysfunction syndrome (MODS) lasting ≥ 7 days compared to those with faster MODS recovery [40]. As a zinc-dependent endopeptidase, MMP9 contributes to extracellular matrix proteolysis and modulates inflammatory cascades via mechanisms involving cytokine activation or inactivation [41,42]. While MMP9’s pro-tumor roles have been documented in other pediatric malignancies—for example, M2-type macrophages in Wilms’ tumor secrete MMP9 to enhance the epithelial–mesenchymal transition (EMT) via a PI3K/AKT-dependent pathway, thereby promoting tumor proliferation and metastasis [43]—it also plays a critical role in sepsis. MMP9 had been identified as an immune related gene in severe burns and sepsis [44], and during septic progression, endotoxin-mediated leukocyte hyperactivation drives the concurrent release of proinflammatory mediators and concomitant MMP9 overexpression [45]. In relapsed B-ALL, MMP9’s pathogenic roles are equally prominent. Experimental evidence indicates MMP9-mediated NF-κB signaling pathway activation induces drug resistance gene expression in ALL cells, exacerbating therapeutic refractoriness [46,47]. Additionally, MMP9 in the bone marrow microenvironment (BMM) reduces degradation of the extracellular matrix protein and impairs B-ALL cell invasion; pharmacological inhibition of MMP9 significantly prolonged survival in mice with B-ALL, indicating that MMP9 may act as an adjunctive therapeutic target for B-ALL [48]. Importantly, MMP9 secretion has also been identified as an independent prognostic factor in childhood B-ALL [49]. Collectively, these shared mechanisms suggest that LTF and MMP9 may function as pivotal molecules connecting inflammation, immune responses, and tissue remodeling. Their synergistic roles in both pediatric sepsis and relapsed B-ALL reflect underappreciated shared pathophysiological features between these two conditions. To our knowledge, this study is the first to identify a link between LTF, MMP9 and B-ALL recurrence. Subsequent investigations warrant mechanistic clarification of these molecule regulators across distinct disease contexts, coupled with systematic evaluation of their therapeutic applicability when targeted in combinatorial approaches.

The current investigation employed projected TFs and miRNAs to build regulatory networks for functionally relevant shared genes. Specificity proteins (Sp), members of the Sp/Kruppel-like factor family, demonstrate elevated expression patterns across various tumor and cancer cell lineages, with particularly pronounced expression observed for Sp1, Sp3, and Sp4. Research by Chen et al. suggested that Sp1 transcription factor alleviates cardiac damage caused by sepsis via Zinc Finger NFX1-Type Containing 1 (ZNFX1) antisense RNA 1 (ZFAS1)/Notch signaling in H9C2 cells [50]. Stephen Safe et al. posited that anticancer agents inducing reactive oxygen species first lead to the downregulation of Sp1, Sp3, and Sp4, predominantly present in solid tumor-derived cells [51]. Moreover, Chen et al. established ETS1 and SP1 as transcriptional regulators of DEAH-box helicase 15 (DHX15) in ALL [52]. These findings collectively support the proposed mechanistic involvement of Sp1 in both sepsis pathogenesis and relapsed B-ALL progression through LTF and MMP9-mediated pathways.

The polyphenolic compound curcumin, derived from Curcuma longa rhizomes, exhibits well-characterized therapeutic properties including potent anti-inflammatory, antioxidant, and anticancer properties. Experimental data from Tong et al. demonstrate that curcumin-mediated AMP-activated protein kinase (AMPK) activation inhibits metastatic progression in colon carcinoma through transcriptional suppression of p65 NF-κB, urokinase plasminogen activator (uPA), and MMP9 [53]. In hematologic oncology [54], curcumin has been shown to reduce survival rates and inhibit proliferation in leukemia [55], myeloma [56], and lymphoma cells [57]. Furthermore, it enhances drug sensitivity in hematologic malignancies, particularly myeloma. Reserpine, an older medication originally used for managing hypertension, creates a competitive blockage of the transport of monoamine into presynaptic storage vesicles by interacting with vesicular monoamine transporter type 2 (VMAT2) as an internal monoamine. While Verma et al. [58] have identified therapeutic efficacy of curcumin in metabolic syndrome management, it is critical to clarify that the findings related to curcumin and reserpine in our study are purely in silico predictions derived solely from molecular docking simulations using AutoDockvina. These docking results-including the high-affinity interactions between MMP9 and curcumin (−7.18 kcal/mol) as well as LTF and reserpine (−5.4 kcal/mol)-have not been validated by prior experimental evidence (e.g., in vitro binding assays or in vivo models) and remain theoretical. Regarding pharmacokinetic differences and safety considerations, curcumin and reserpine have partial adult pharmacokinetic data, but their pediatric ADME needs clarification. Children’s unique physiological features (immature hepatic cytochrome P450 enzymes, underdeveloped renal clearance, distinct body composition) may cause differences in drug bioavailability, half-life, and metabolites vs. adults, and pediatric-specific pharmacokinetic studies are needed for age-appropriate dosing balancing efficacy and risk [59,60]. Safety is more pressing: pediatric data on these drugs is limited, and children’s developing immunity, vulnerable organs, and higher ADR susceptibility increase toxicity risk. Thus, rigorous preclinical evaluations (e.g., age-stratified animal models) and phase I/II trials, along with long-term follow-up studies to assess potential impacts on children’s growth, development, and organ function, are essential to define dose-limiting toxicities, ADR profiles, and optimal durations before pediatric clinical use [61].

While this study provides initial evidence for the association between LTF and MMP9 gene expression across both target diseases through RT-qPCR analysis, and computational docking simulations indicate possible therapeutic utility of curcumin and reserpine, several limitations warrant acknowledgment. Firstly, RT-qPCR validation only included 5 sample pairs—this small size may cause large standard errors and low statistical power, limiting result reliability and generalizability [62]. Future work will recruit more samples across regions to enable large-scale validation and improve statistical significance/reproducibility. Secondly, the current investigation only validated LTF and MMP9 expression at the transcriptional level, with no verification at the protein level. Differences between mRNA and protein expression may arise due to factors such as protein synthesis, degradation, and post-translational modification [63]. In subsequent analyses, we will use Western blot to detect the protein expression levels of LTF and MMP9 and, where applicable, employ ELISA to quantify related secreted proteins. Third, we recognize the inherent limitations of computational docking (reliance on static structures, simplified scoring). Although curcumin and reserpine show theoretical therapeutic potential for pediatric sepsis and relapsed B-ALL, their translation into pediatric clinical practice is premature. Future work will prioritize biochemical experiments to confirm drug-target (LTF/MMP9) binding, preclinical studies of their functional effects on pathways related to sepsis/relapsed B-ALL, determination of pediatric-specific pharmacokinetics profiles, and establishment of safety/tolerability in pediatric cohorts. Fourth, the lack of multiple testing correction increases the risk of type I errors (false positives), and the study is limited to transcriptional profiling, lacking experimental validation of LTF and MMP9 functional roles in IL-17 signaling pathways.

Future research efforts should focus on two critical directions: methodological enhancement through larger cohort analyses and multi-center independent validation to comprehensively evaluate the clinical value of the diagnostic methodology, coupled with rigorous multiple comparison adjustments (Bonferroni correction or False Discovery Rate) for improved result robustness. Additionally, deeper exploration into the functional mechanisms of the coregulated genes and drug interactions is warranted, utilizing techniques including Western blot, animal models, and gene editing. Further investigation using single-cell analysis and flow cytometry should focus on revealing their expression patterns within immune cells and elucidating their immunoregulatory roles. This multifaceted approach will provide a solid foundation for clinical translation.

Finally, while existing studies have confirmed an association between sepsis and relapsed B-ALL, the specific causal mechanism (including the direction of interaction) between the two conditions still requires further experimental investigation, we plan to explore the association between pediatric sepsis and ALL recurrence through genetic association analysis based on cross-sectional data and longitudinal cohorts.

## 5. Conclusions

In conclusion, this study revealed the potential molecular mechanisms of key common genes with LTF and MMP9 in pediatric sepsis and relapsed B-ALL, which could provide novel insights for the clinical diagnosis of these two diseases. Despite the potential therapeutic benefits of *Curcumin* and *Reserpine*, their use in clinical practice in pediatric sepsis and relapsed B-ALL is still in the investigational phase, and more clinical trials and studies are needed to assess their safety and efficacy.

## Figures and Tables

**Table 1:** The sequences of the target primers.

Primer	Sequence
LTF-F	5′-ATGGTGGTTTCATATACGAGGCA-3′
LTF-R	5′-CTTTCGGTCCCGTAGACTTCC-3′
MMP9-F	5′-TGTACCGCTATGGTTACACTCG-3′
MMP9-R	5′-GGCAGGGACAGTTGCTTCT-3
GAPDH-F	5′-GGAGTCCACTGGCGTCTTCA-3′
GAPDH-R	5′-GTCATGAGTCCTTCCACGATACC-3′

## Data Availability

The datasets analyzed during the current study are available in the GEO (https://www.ncbi.nlm.nih.gov/geo/) (accessed on 2 September 2023) [GSE13904, GSE80496, GSE3910 and GSE28460] repository, further inquiries can be directed to the corresponding author (CL).

## Supplementary Materials

The following supporting information can be downloaded at: https://www.mdpi.com/article/10.3390/biomedicines13092307/s1, Figure S1: Protein-protein interaction (PPI) network among common DEGs; Figure S2: Network plots of the TF-mRNA and miRNA-mRNA; Figure S3: The expression of LTF and MMP9; Table S1: Lists of 164 common genes of children sepsis and ALL; Table S2: Results for GO enrichment terms of 164 common genes; Table S3: Results for KEGG enrichment pathways of 164 common genes.

## Abbreviations

ALL: acute lymphoblastic leukemia; B-ALL: B-cell lineage ALL; HSCT: hematopoietic stem cell transplantation; CAR: chimeric antigen receptor; GEO: Gene Expression Omnibus; HC: healthy control; DEGs: Differentially expressed genes; GO: Gene Ontology; KEGG: Kyoto Encyclopedia of Genes and Genomes; SVM-RFE: support vector machine recursive feature elimination; ROC: receiver operating characteristic; TFs: transcription factors; miRNAs: microRNAs; DGIdb: Drug-Gene Interaction database; ceRNA: competing endogenous RNA; LTF: lactotransferrin; MMP9: matrix metallopeptidase 9; RT-qPCR: Real time quantitative PCR.
