# Supplementary material for: Key Common Genes with LTF and MMP9 Between Sepsis and Relapsed B-Cell Lineage Acute Lymphoblastic Leukemia in Children
Source: Biomedicines. 2025 Sep 20;13(9):2307. doi: 10.3390/biomedicines13092307 (PMC12467856; doi:10.3390/biomedicines13092307)
Supplement: Supplementary file 1 [file biomedicines-13-02307-s001.zip › Figures S1-S3.pdf]

Supplemental Materials

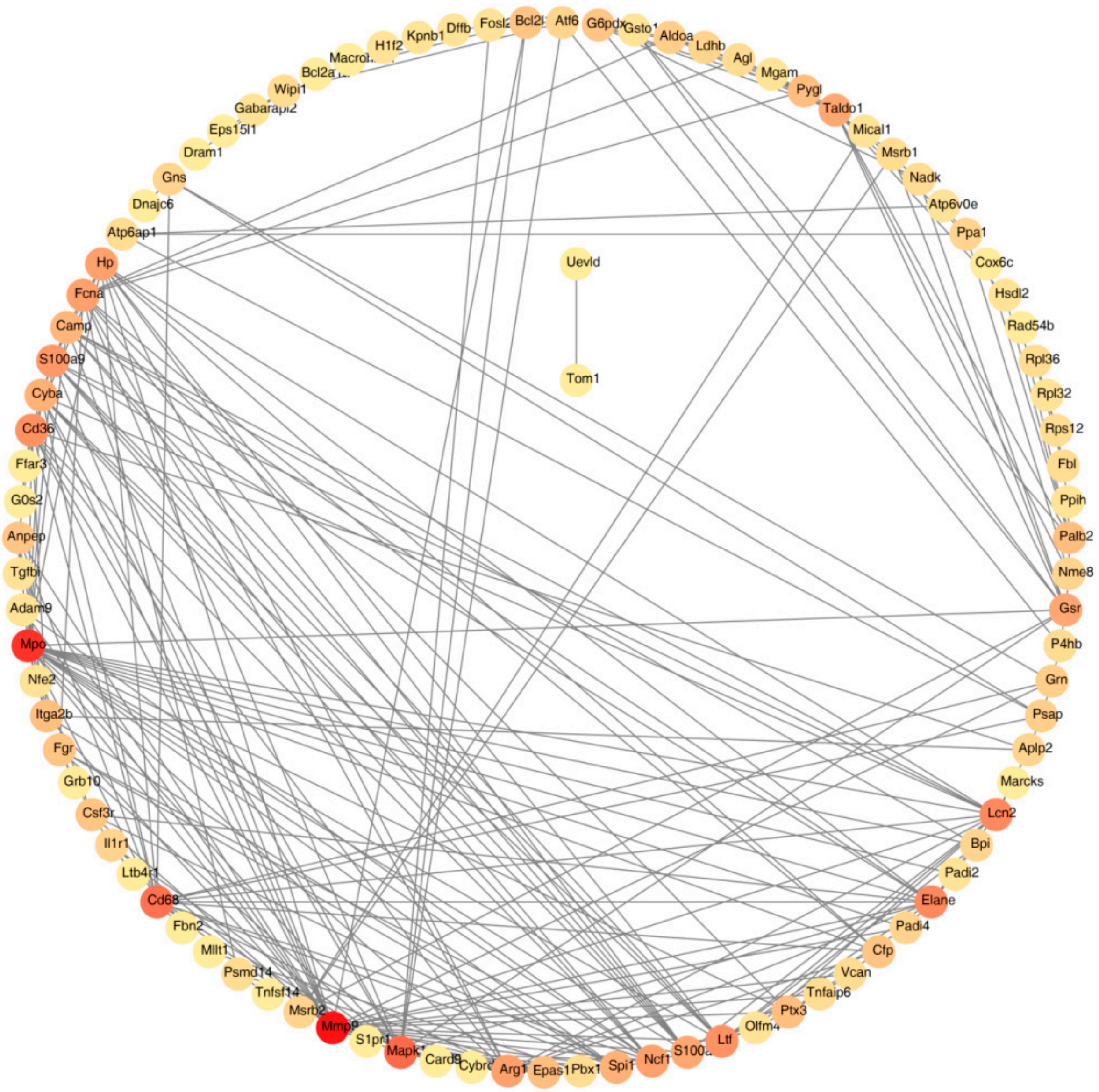

Figure S1. Protein-protein interaction (PPI) network among common DEGs.

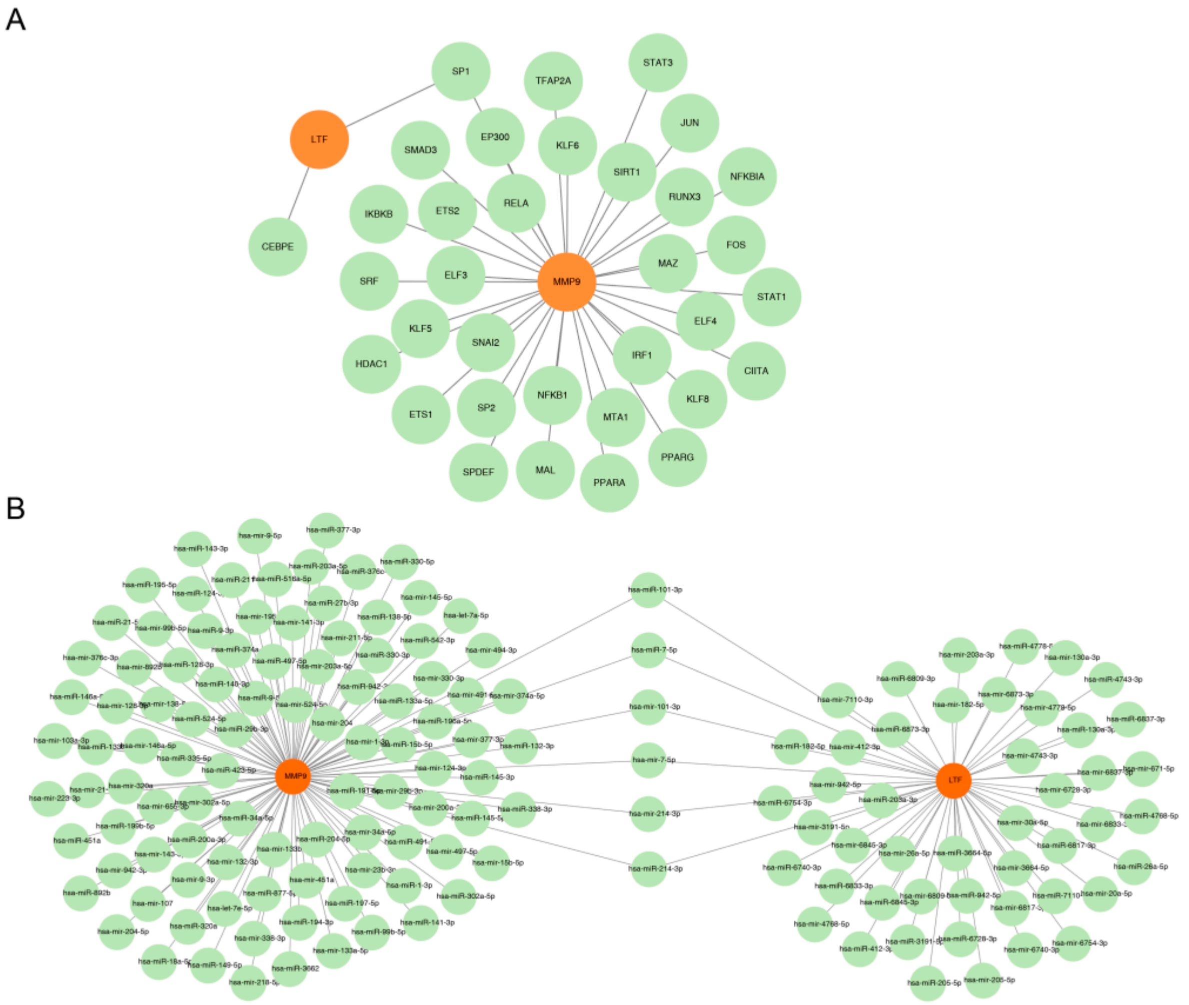

Figure S2. Network plots of the TF-mRNA and miRNA-mRNA. (**A**) TF-mRNA network diagram, green node: transcription factor; red node: Hub gene. (**B**) miRNA-mRNA network diagram of Hub gene, green node: miRNA; red node: Hub gene.

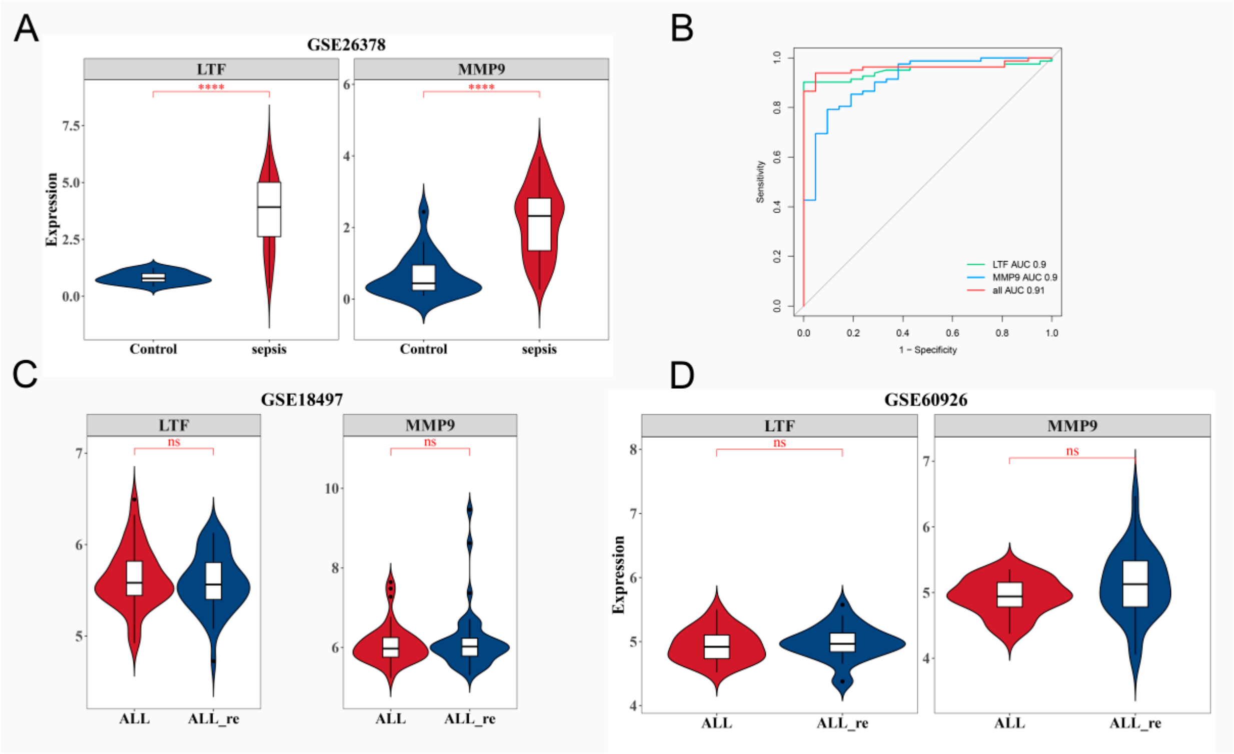

Figure S3 The expression of LTF and MMP9: (**A**) The expression of LTF and MMP9 in the GSE26378; (**B**) The roc curve of LTF and MMP9 in the GSE26378; (**C**) The expression of LTF and MMP9 in the GSE18497; (**D**) The expression of LTF and MMP9 in the GSE60926

Titles for Supplemental tables

Table S1. Lists of 164 common genes of children sepsis and ALL.

Table S2. Results for GO enrichment terms of 164 common genes.

Table S3. Results for KEGG enrichment pathways of 164 common genes.
